# Updates in imaging in ocular oncology

**DOI:** 10.12688/f1000research.19979.1

**Published:** 2019-10-01

**Authors:** Jose R. Davila, Prithvi Mruthyunjaya

**Affiliations:** 1 Ophthalmology, Stanford Byers Eye Institute, Palo Alto, CA, 94303, USA

**Keywords:** Ocular oncology, melanoma, retinoblastoma, radiation retinopathy, ocular surface squamous neoplasia, optical coherence tomography, angiography

## Abstract

Innovations in ophthalmic imaging have made a profound impact on the diagnosis and treatment of ophthalmic disease. In ocular oncology, the development of optical coherence tomography with enhanced depth imaging and swept source technologies has made it possible to visualize the anatomical characteristics of retinoblastoma and uveal melanoma with a level of detail previously unobtainable on clinical exam alone. As a result, our understanding of the pathophysiology of vision loss in choroidal melanoma in particular has improved. These modalities have also helped identify fundoscopically “invisible” tumors and risk stratify pre-malignant choroidal lesions, making a strong case for their inclusion in all screening evaluations. Optical coherence tomography angiography, on the other hand, has allowed non-invasive imaging of the retinal and uveal vasculatures, providing insight into vascular changes associated with malignant transformation and vision loss following exposure to radiation. While the impact of new imaging technologies on clinical outcomes and overall survival in ocular oncology has yet to be determined, several reports cited herein offer promising results.

## Introduction

Recent advances in imaging have made a profound impact on the diagnosis and treatment of both anterior and posterior segment ophthalmic disease. The field of ocular oncology is no exception to this trend; novel imaging modalities are enhancing our understanding of intraocular tumors, their impact on ocular physiology, and their response to treatment. The utility of fundus photography and ultrasound (US) imaging has been magnified over the past few years by the adjunctive use of newer modalities – in particular, new forms of ocular coherence tomography (OCT) and OCT angiography (OCTA). The present review serves as a brief update on these imaging modalities and their current role in the characterization of choroidal melanoma (CM), retinoblastoma, anterior segment tumors, and radiation retinopathy.

## Choroidal melanoma

CM is the most common primary intraocular malignancy in adults. Historically, choroidal nevi and melanomas have been diagnosed and monitored with fundoscopic examination, fundus photography, and ocular US. Invasive methods, such as fluorescein angiography (FA) and indocyanine green angiography (ICGA), have been used where necessary to help differentiate melanoma from masquerading pathology, to assess for secondary neovascularization or ischemia, or to assist in visualizing lesions obscured by media opacity ^1^. More recently, OCT and OCTA have made it possible to non-invasively image superficial and deep structures of the retina and choroid with a level of detail previously unobtainable by clinical exam alone.

Enhanced depth imaging (EDI) OCT using spectral domain (SD) technology and swept source (SS) OCT, with faster scanning engines, are technologies employed to image deep intraocular structures from the choroid to the inner sclera. While EDI SD-OCT is an adaptation of prior technology, SS-OCT is a newer technology that uses a longer wavelength (1,050 nm compared to 840 nm), reduced sensitivity roll-off, and faster scanning speeds, allowing for better light penetration through the RPE, higher likelihood of detecting subtle signals from deeper layers, and denser scan patterns over larger scan areas ^2^. Both imaging technologies offer a superior level of detail of the deep intraocular structures when compared to ocular US. One recent study highlighting the power of EDI-OCT brings to light five distinct imaging patterns of flat choroidal nevi that were previously not appreciated with US alone ^3^. Of 94 fundoscopically and ultrasonographically flat nevi included in this review, nearly one-third were found to be elevated on EDI-OCT ^3^. Interestingly, only nevi demonstrating elevation on initial EDI-OCT were found to show growth or change over a 22 month follow-up time period ^3^. Furthermore, the posterior lesional border was visible in a majority of cases (approximately 80%), making it possible to monitor for multi-dimensional change ^3^. In a second recent study on EDI-OCT, Shield *et al.* found that the thickness of small CM was more accurately determined by EDI-OCT as compared to US, which may overestimate size by up to 55% ^4^. Both EDI-OCT and SS-OCT have been used to characterize CM and associated changes in the overlying neurosensory retina. Characteristic features of melanoma include deep optical shadowing, thinning or compaction of the choriocapillaris, disruption of the adjacent retinal photoreceptor layer, presence of subretinal fluid with or without lipofuscin deposition, and presence of intraretinal fluid ^4^.

**Figure 1.**
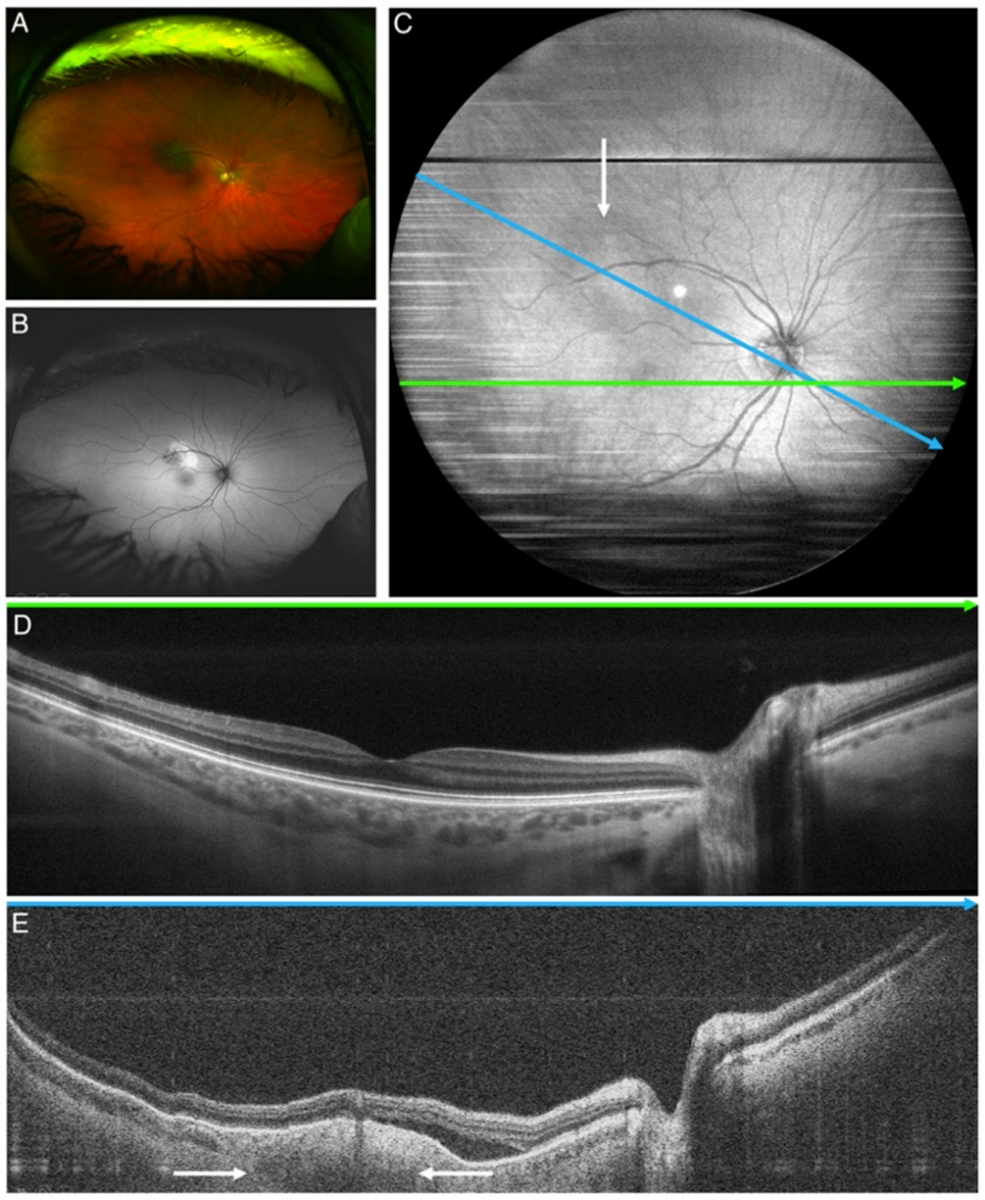
Wide field of view image of choroidal nevus (white arrows). Images used with permission from Mcnabb *et al*. ^7^. **A**) Red/green and **B**) autofluorescence scanning laser ophthalmoscopy. **C**) Wide field of view swept-source optical coherence tomography (OCT) B-scan projection, blue line corresponding to position of image **D**), which demonstrates normal optic nerve, fovea, and retinal layers. **E**) OCT corresponding to green arrow demonstrating choroidal nevus with overlying drusen, loss of choriocapillaris, and associated subretinal fluid.

**Figure 2.**
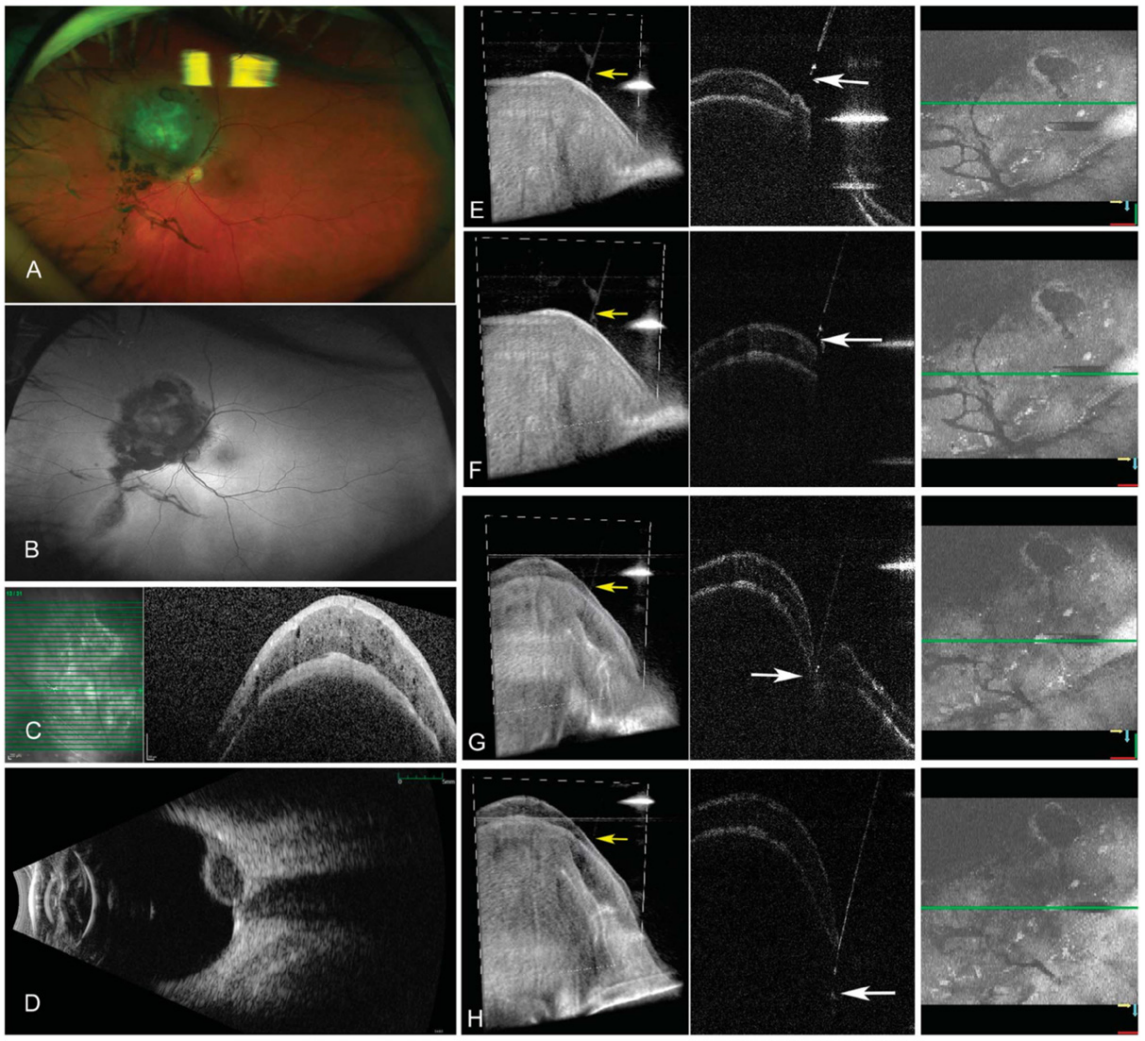
Intraoperative 4D-microscope-integrated optical coherence tomography (4D-MIOCT). Images used with permission from Grewal *et al*. ^8^. **A**) Elevated choroidal lesion superonasal to optic nerve without surrounding hyperautofluorescence ( **B**), consistent with choroidal melanoma. **C**) OCT showing choroidal tumor without subretinal fluid. **D**) Ultrasound showing low internal reflectivity. **E**– **H**) sequential images from intraoperative 4D-MIOCT with three-dimensional volumes showing cutter tip making contact with and penetrating the retinal surface at nearly perpendicular orientation with adequate depth ( **H**) (white and yellow arrow show cutter tip).

Recently, SS-OCT has been modified algorithmically to produce “speckle noise-free” images, which are meant to reduce the granular noise signal inherent to several imaging methods including OCT, computerized tomography, and magnetic resonance ^5, 6^. OCT technology has also been adapted to view peripheral tumors. In one report, SS-OCT was modified with wide-angle, 20 and 40 diopter indirect ophthalmoscopy lenses to capture wide field-of-view images of peripheral CM in a single imaging session ( Figure 1) ^7^. The field of view was a mean of 4.6 optic disc-to-fovea distance units compared to the mean 2.1 disc-to-fovea units of SD-OCT and 9.4 disc-to-fovea units of widefield scanning laser ophthalmoscopy ^7^. Furthermore, microscope-integrated OCT (MIOCT) has been used intra-operatively to guide real-time (4D-MIOCT) 27-gauge transvitreal biopsy of CM ( Figure 2) ^8^. The reported benefit of intra-operative 4D-MIOCT is to achieve adequate biopsy depth with the vitrectomy cutter, avoid unnecessary damage to surrounding neurosensory retina, and avoid the risk of deep penetration and subsequent choroidal hemorrhage.

The advent of OCTA has given further insight into retinal vascular abnormalities induced by CM. Specifically, OCTA has been able to non-invasively demonstrate a significant enlargement of the deep foveal avascular zone and a decrease in superficial and deep parafoveal capillary vascular densities in eyes with CM as compared to their fellow healthy eyes ^9^. These changes appear to be correlated with the presence of subretinal fluid and larger tumor size, bringing to light the possible role of tumor-related pro-inflammatory factors, such as vascular endothelial growth factor (VEGF), in the pathogenesis of vision loss in CM ^9^. One proposed mechanism behind the association between CM size and presence of subretinal fluid is a VEGF-induced microvascular compromise that precedes macular edema in affected eyes ^9^. Furthermore, these features appear to be absent in eyes with choroidal nevi, giving them a potential role in differentiating choroidal nevi from melanoma ^10^.

While retinal vascular imaging is well suited to OCTA, imaging of the deeper choroidal vessels remains a challenge, both in the posterior pole and in the choroidal periphery. ICGA, which involves injection of intravenous dye, is the standard for choroidal vascular imaging. More recently, a novel method for OCTA, SS-OCTA, has shown the capacity to non-invasively visualize deeper choroidal vessels with a level of detail akin to that of ICGA ^11^. In one case report, it was used to diagnose choroidal neovascularization arising from a choroidal nevus masquerading as neovascular age-related macular degeneration ^12^. While not a usual part of the pre- or post-treatment evaluation of CM, routine, non-invasive imaging of the choroidal vasculature may reveal microvascular patterns that have previously been implicated in higher degrees of malignancy, metastasis, and successful eradication after radiation therapy ^13, 14^. Choroidal vascular features observed to potentially differentiate nevus from melanoma include irregularity of lesion margins, heterogeneous hyporeflective choriocapillary plexus with avascular areas, hyperreflective choriocapillary rings, thick choroidal vascular networks, and choroidal vascular loops ^15, 16^. In a study by Fuste *et al.* comparing OCTA findings of 18 cases of choroidal nevus to 18 cases of choroidal melanoma, border irregularity, choriocapillaris hyporeflectivity, presence of avascular areas, and vascular anomalies, such as thick networks or vascular loops, were found to be present in a majority of cases of CM but only a minority of choroidal nevi ^15^. Interestingly, one of the 18 cases of CM treated with brachytherapy demonstrated complete regression of vascular anomalies seen prior to treatment ^15^.

The malignant potential of a presumed choroidal nevus is most accurately assessed by considering key features from several different imaging modalities. In a recent study by Shields *et al.* ^17^, over 2,000 cases of choroidal nevus with multimodal imaging were reviewed to assess for features imparting a higher risk of development of malignant features. Significant risk factors included US thickness >2 mm, subretinal fluid on OCT, central visual acuity loss, orange pigment on autofluorescence, lesion hollowness (US), and diameter >5 mm on photography. The presence of a greater number of risk factors was found to correlate with a greater risk of the development of malignant features. At 5-year follow-up, the percentage of lesions that transformed into melanoma were 1% (hazard ratio [HR] 0.8) for no features, 11% (HR 3.09) for one feature, 22% (HR 10.6) for two features, 34% (HR 15.1) for three features, 51% (HR 15.2) for four features, and 55% (HR 26.4) for five features. It will be interesting to see the role of newer imaging modalities, such as OCTA, in the evaluation of high-risk choroidal nevi.

## Retinoblastoma

Retinoblastoma is the most common primary intraocular malignancy in children worldwide, with an estimated incidence of one in 15,000 to 18,000 live births ^17^. Recent advances in ophthalmic imaging have improved our understanding of the anatomy of retinoblastoma, its relationship to the fovea, and, thus, implications on visual prognosis *in vivo*. SD-OCT and hand-held SD-OCT (HHSD-OCT), the latter performed bedside during examination under anesthesia, have made it possible to evaluate the anatomical position of tumors with a new level of precision ^18– 21^. Retinoblastoma has been characterized on OCT as a retinal tumor arising from inner, middle, or outer retinal layers with disorganization of surrounding retina and with occasional intralesional cavities ^22^. “Retinal draping” is a term used to refer to the outer retinal tumor with overlying normal-appearing inner retinal layers ^23^. Using OCT, it is possible to visualize the border between anatomically normal- and abnormal-appearing retina, and to evaluate the structural integrity of the fovea, and thus to estimate the possible impact on visual potential ^24, 25^.

**Figure 3.**
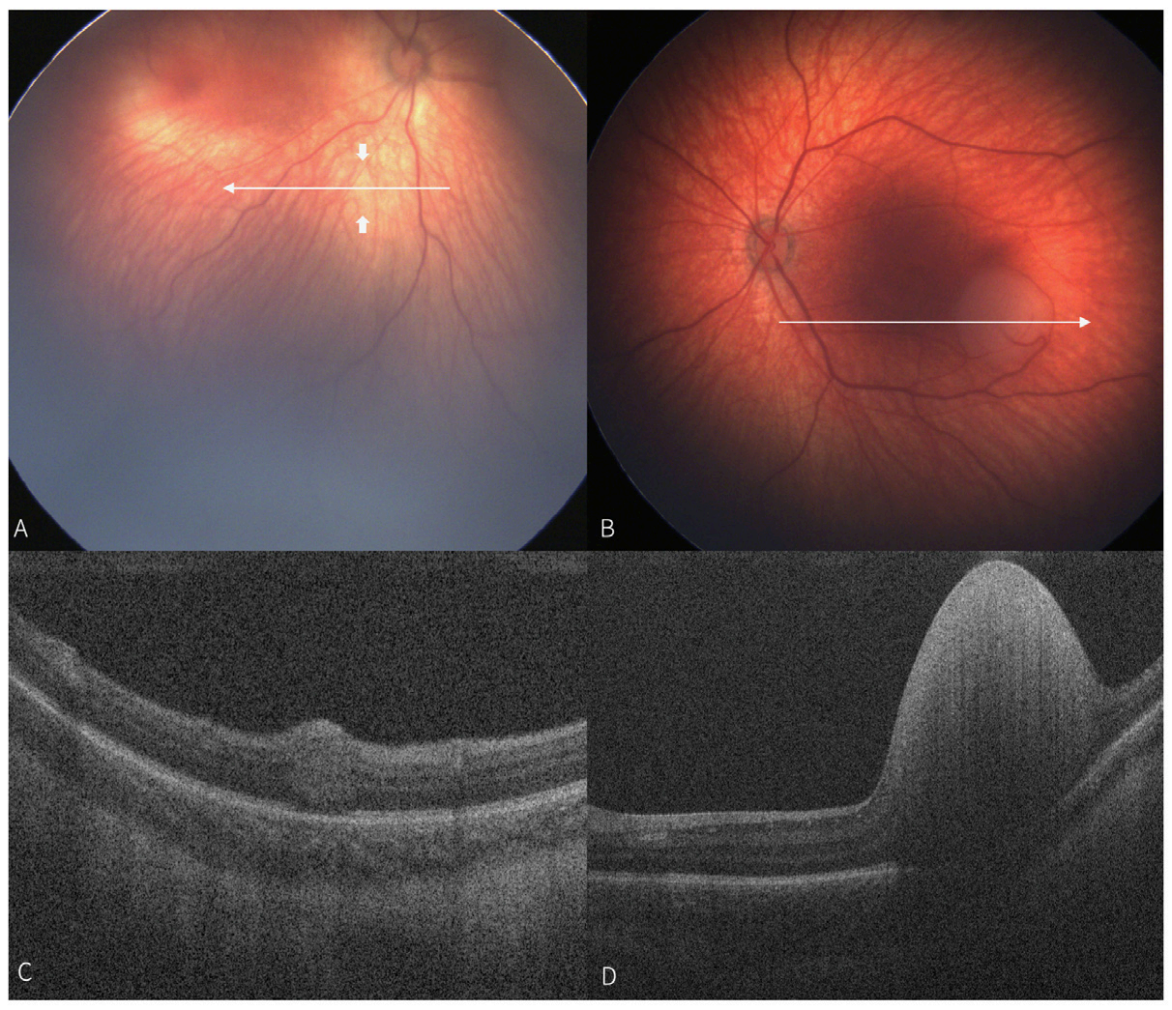
Fundoscopically invisible retinoblastoma. Written informed consent was obtained from the patient's legal guardian in Figure 3, for the use and publication of these images. **A**) Fundus photograph of a right eye taken during examination under anesthesia, arrowheads pointing location of transparent retinal mass seen centrally in image **C**. **B**) Fundus photograph of left eye taken during examination under anesthesia, with large, elevated retinoblastoma inferotemporal to fovea. **C**) Optical coherence tomography (OCT) of right eye demonstrating retinal mass not visualized on fundoscopy (located between arrows in image **A**). **D**) OCT of left eye demonstrating large, elevated hyperreflective retinal mass with loss of normal retinal layers, corresponding to mass visualized in image **B**.

As in other retinal diseases, OCT has the potential for improving diagnostic accuracy, clinical staging, and treatment outcomes in retinoblastoma. In a recent retrospective review of 63 eyes from 44 children diagnosed with retinoblastoma and with a median of five HHSD-OCT sessions per eye, Soliman *et al*. found that OCT changed the diagnosis (excluding tumor in clinically suspicious areas, changing staging group, or detecting clinically invisible tumor) in seven out of 293 (3%) OCT sessions, changed treatment (detection of unsuspected tumor within scar) in 27 out of 293 (11%) cases, and changed follow-up (showing anatomical details excluding activity and thereby changing treatment plan) in 16 out of 293 (7%) OCT sessions ^26^. There are now several reports of HHSD-OCT being used to detect submillimeter and fundoscopically “invisible” tumors ( Figure 3). Specifically, OCT has been used to detect and monitor response to treatment in cases of parafoveolar submillimeter retinoblastoma and retinoblastoma resistant to chemotherapy ^27– 30^. In cases of “invisible” tumors, HHSD-OCT has been able to detect tumor activity in the contralateral eye of patients with known unilateral retinoblastoma and in a patient with strong family history of retinoblastoma undergoing screening evaluation ^31– 33^. The long-term impact of these new imaging modalities on life expectancy and visual outcome is yet to be discovered.

## Anterior segment tumors

**Figure 4.**
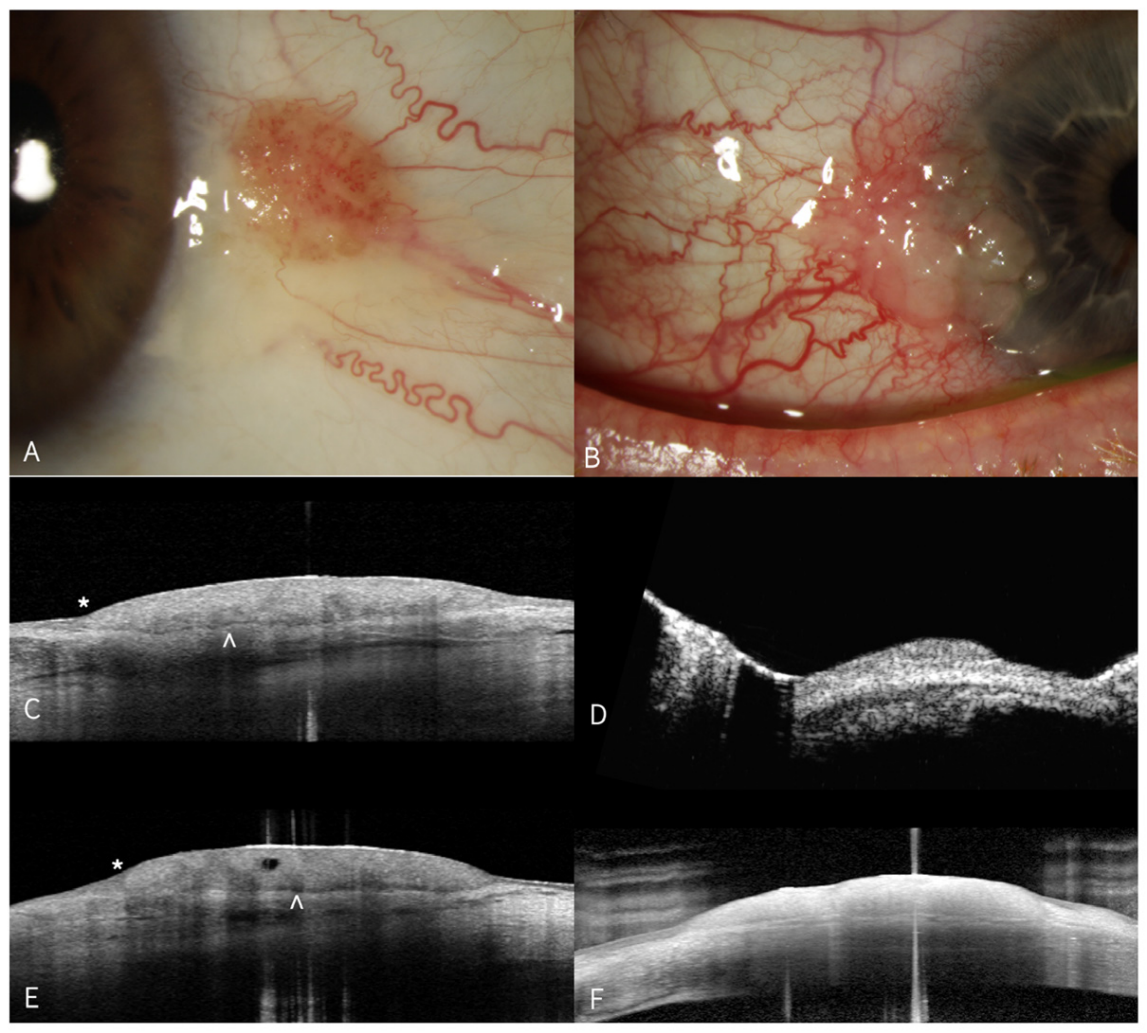
Ocular surface squamous neoplasia (OSSN). Written informed consent was obtained from the patient in Figure 4, for the use and publication of these images. **A**) External photograph of left eye showing OSSN with large feeder vessel and surrounding pinguecula temporal to limbus. **C**) Horizontal and **E**) vertical anterior segment (AS) OCT scans demonstrating abrupt transition to elevated epithelial lesion (asterisk) and clear plane of separation from underlying tissue (white arrow). **B**) External photograph of a right eye with OSSN lesion extending from temporal limbus onto cornea. AS-OCT of the lesion ( **F**) demonstrates significantly higher resolution than ultrasound biomicroscopy of the same lesion ( **D**).

Ocular surface squamous neoplasia (OSSN) refers to several distinct histopathologic entities of the conjunctiva and cornea, ranging from dysplasia to invasive carcinoma ^34^. OSSN has traditionally been diagnosed and monitored by slit lamp examination, serial slit lamp photography, and US biomicroscopy (UBM). The advent of high-resolution OCT (HR-OCT) and ultra-HR OCT (UHR-OCT) of the anterior segment, attaining a resolution of less than 5 microns, has introduced the possibility of evaluating lesions obscured from external evaluation due to overlying ocular surface disease ^35^. In one study by Atallah *et al*., biopsy-confirmed OSSN obscured by overlying ocular surface degeneration and/or scarring (due to rosacea, pterygia, Salzmann nodular degeneration, or limbal cell deficiency with scarring) was detected reliably with HR-OCT ^36^. The primary lesional characteristics seen in cases of OSSN were thickening of the epithelial layer and an abrupt transition of epithelial reflectivity between normal and abnormal tissue ^36^. A separate study using time domain anterior segment OCT found 90% of OSSN cases to exhibit a clear hyporeflective plane of separation between thickened epithelium and underlying tissue ( Figure 4) ^37^. Still, OCT has limitations. In a direct comparison between UBM and OCT imaging of conjunctival nevi, UBM demonstrated superiority in visualizing lesions that were highly pigmented and remarkably elevated. OCT, on the other hand, was more sensitive for visualizing the internal structures of nevi, such as intralesional cysts ^38^.

OCTA has been applied to visualize malignant iris melanomas in comparison to benign iris lesions, such as iris freckles, nevi, and pigment epithelial cysts. In one cross-sectional study, OCTA of iris melanomas demonstrated vascular tortuosity and disorganization with increased intralesional vascular density as compared to benign iris lesions and normal controls ^39^. These findings were previously described using FA ^40^. Interestingly, lesions treated with radiation therapy demonstrated a subsequent reduction in intralesional vascular density ^39^.

## Radiation retinopathy

Radiation retinopathy is a chronic vascular retinopathy that develops after exposure to ionizing radiation from radioactive plaque use for the treatment of intraocular tumors or external beam radiation for head and neck cancers. The underlying pathophysiology shares similarities with rapidly progressive diabetic retinopathy. Certain patients appear to have a higher likelihood of developing radiation retinopathy. Specifically, those with pre-radiation subclinical macular edema, with large tumors, with tumors close to the foveola, and with tumors associated with overlying hemorrhage ^41^. As demonstrated by Rose *et al*. using prototype doppler SD-OCT and hyperspectral retinal camera, total retinal blood flow and retinal blood oxygen saturation were significantly lower in patients with unilateral ischemic radiation retinopathy as compared to their healthy fellow eye ^42^. Early vascular changes may be first detected with OCTA, as irregular widening of the foveal avascular zone, discontinuity of retinal vasculature, and microaneurysms of the deep or superficial retina ^43^. These features, in combination with SD-OCT findings, including cystic macular edema and increased macular thickness, have been used to generate a new grading scheme for radiation retinopathy as well as to guide treatment with intravitreal anti-VEGF agents ^43, 44^.

## Conclusions

Recent innovations in ophthalmic imaging have made a significant impact on our understanding of ocular oncology and its effect on ocular physiology. Given their potential for detecting early disease with high sensitivity, it will be interesting to see the impact they may have on visual prognosis and life expectancy.
